# Current Evidence of Chinese Herbal Constituents with Effects on NMDA Receptor Blockade

**DOI:** 10.3390/ph6081039

**Published:** 2013-08-21

**Authors:** Willmann Liang, Wai Ping Lam, Hong Chai Tang, Ping Chung Leung, David T. Yew

**Affiliations:** Institute of Chinese Medicine, The Chinese University of Hong Kong, Hong Kong; E-Mails: iceicemy@cuhk.edu.hk (W.P.L.); b119594@cuhk.edu.hk (H.C.T.); pingcleung@cuhk.edu.hk (P.C.L.)

**Keywords:** NMDA receptor, antagonist, herb, Chinese medicine

## Abstract

NMDA receptor (NMDA-R) is an important molecular entity governing a wide range of functions in the central nervous system. For example, the NMDA-R is involved in memory and cognition, and impairment of both (as in Alzheimer’s Disease) is attributed to NMDA-mediated neurotoxicity. With greater understanding of the NMDA-R structure, antagonists with varying degrees of binding-site and subtype selectivity have been developed and put into clinical use. Discovery of target-specific Chinese herbs have also been made in parallel. This article provides an overview of the known active sites on the NMDA-R, followed by a discussion of the relevant herbs and their constituents. Experimental evidence supporting the inhibitory role of the herbal compounds on the NMDA-R is highlighted. For some of the compounds, potential research directions are also proposed to further elucidate the underlying mechanisms of the herbs. It is envisaged that future investigations based on the present data will allow more clinically relevant herbs to be identified.

## 1. Introduction

Glutamate is an excitatory central neurotransmitter that activates mainly three ionotropic receptor subtypes: AMPA, NMDA and kainate, in addition to a metabotropic subtype [1]. Activation of the NMDA receptor (NMDA-R) results in generation of slow excitatory postsynaptic potentials that govern a variety of brain functions, most notable of which is long-term potentiation in the consolidation of long-term memory [1]. Since NMDA-R is itself an ion channel permeable to Ca^2+^ (and also Na^+^) entry, prolonged receptor activation may lead to pathological consequences such as Ca^2+^ overload, oxidative stress, mitochondrial damage and eventually cell death [1]. Strong evidence points to NMDA-induced excitotoxicity manifested in neurodegenerative diseases and neuronal damages following cerebral ischaemia [1,2,3,4]. Pain transmission is also dependent on glutamate and NMDA-R has been associated with neuropathic pain [4,5]. Other neuronal disorders such as major depression [6] and epilepsy [7] are also attributed to abnormal NMDA-R activity. NMDA-R antagonists thus present a promising treatment strategy for many of the conditions above, as evidenced by the development of novel, site-specific drugs over the years [8].

The NMDA-R has multiple modulatory sites [2,9] that serve as targets of conventional receptor antagonists. The agonist-binding domain (ABD) of NMDA-R is located on a tetramer of two NR2 and two NR1 subunits [2]. Traditionally the NR2 subunit, which has a glutamate-binding site, and the channel pore have been the subject of research. One of the first NMDA-R antagonists, D-aminophosphonovaleric acid (APV), acts as a competitive antagonist at the glutamate-binding site [2]. In view of the serious side effects usually accompanying nonselective, competitive NMDA-R antagonism, recent effort has been directed toward the development of antagonists at the N-terminal domains of the NR2 (and NR1) subunits. For example, ifenprodil and related compounds have shown NR2B-blocking effects [10] while other NR2A- [11,12,13], NR2C- [14] and NR2D-selective antagonists [15] have also been identified [12,14,15]. Unlike blockers at the glutamate-binding site, the NR2-selective compounds act as noncompetitive antagonists and the effects of some are affected by glycine but not glutamate [2,8]. A few compounds acting at the glycine-binding site (on the NR1 subunit) are also under development [8]. It should be noted that despite the improved selectivity, these newer compounds only display partial antagonistic activity [9]. Some NR2-subtype-selective antagonists also show NR1-blocking effects, as exemplified by Zn^2+^ (NR2A-selective) and ifenprodil (NR2B-selective) [13,16,17,18]. Another classical drug target site is the channel pore, with dizocilpine (MK-801) and polycyclidine being the prototypical examples [3,9]. The channel blockers are uncompetitive antagonists since they reach the active site only after NMDA-R activation (and channel opening). Clinical application of channel blockers is popular, e.g. memantine is used in Alzheimer’s disease (AD) and ketamine is used as an antidepressant, anaesthetic and analgesic for neuropathic pain [5,19]. In relation to the similar structure between AMPA receptor and NMDA-R, two other drug targets of the latter have been proposed but information is scarce at present. The ABD is located on a dimer of two subunits (a pair of NR1 & NR2 each) and where the dimer interfaces may present a potential regulatory site as shown in the AMPA receptor [9,20]. Secondly, a region connecting the agonist-binding domain with the channel pore, as demonstrated in the AMPA receptor, may be an active site for antagonists [21].

## 2. List of Chinese Herbs with NMDA-R Blocking Effects

From the literature, a number of herbs and their constituents with demonstrated effects in memory, cognition and neuroprotection have not been associated with NMDA-R activity yet. These include those in the Amaryllidaceae family, *Bacopa monnieri*, *Sanguisorba officinalis* and others [22,23]. Examples of these herbs will not be discussed in this review, and only those that modulate NMDA-R activity and/or downstream signaling will be mentioned.

According to the theory and practice of traditional Chinese medicine, herbs are classified based on their therapeutic nature and classical usage, *e.g*. those that promote circulation (“blood-invigorating” herbs) or stabilize mood (“shen-calming” herbs). Interestingly, herbs (or their constituents) that modulate NMDA-R activity are not restricted to any particular category. Table 1 below shall highlight the diversity of these herbs with regards to their classical uses in traditional Chinese medical practice. Also summarized in the table are the demonstrated effects of the herbs on NMDA-R, with reference to the sections of the manuscript where each of the herbs are discussed in greater detail.

**Table 1:** A summary of the herbs and their effects on and related to the NMDA-R.

Clinical usage	Pharmaceutical name	Effect
Herbs that promote circulation (Section 3.1)	Rhizoma Curcumae Longae	Inhibit Ca^2+^ response
	Radix Achyranthis Bidentatae	Inhibit Ca^2+^ response Reduce reactive oxygen species production Inhibit apoptotic enzymes
	Herba Lycopodii Serrati	Block NMDA-induced current
Herbs that promotes blood clotting (Section 3.2)	Radix Notoginseng	Inhibit Ca^2+^ response Reduce reactive oxygen species production Inhibit apoptotic enzymes
Herbs that stabilize mood (Section 3.3)	Radix Polygalae	Inhibit Ca^2+^ response Reduce reactive oxygen species production Reduce excitotoxcity-related cell death
	Semen Zizyphi Spinosae	Inhibit Ca^2+^ response Reduce reactive oxygen species production Reduce excitotoxcity-related cell death
	Radix et Rhizoma Valerianae	Reduce excitotoxcity-related cell death
Herbs with effects on dizziness, headache and seizure (Section 3.4)	Ramulus Uncariae cum Uncis	Block NMDA-induced current Reduce excitotoxcity-related cell death
	Rhizoma Gastrodiae	Reduce excitotoxcity-related cell death Reduce NMDA-induced glutamate release
	Semen Cassiae	Inhibit Ca^2+^ response Reduce excitotoxcity-related cell death
	Rhizoma Acori	Reduce excitotoxcity-related cell death
Herbs that boost the immune system (Section 3.5)	Radix Glycyrrhizae	Inhibit Ca^2+^ response Inhibit apoptotic enzymes Reduce excitotoxcity-related cell death
	Radix Ginseng	Block NMDA-induced current Inhibit Ca^2+^ response Reduce excitotoxcity-related cell death
Herb with antitussive effects (Section 3.6)	Folium Ginkgo	Block NMDA-induced current
Herb with antimicrobial effects (Section 3.7)	Radix Scrophulariae	Inhibit Ca^2+^ response Reduce reactive oxygen species production Reduce excitotoxcity-related cell death

## 3. Experimental Evidence of Individual Herbs in Modulating NMDA-R Activity

### 3.1. Rhizoma Curcumae Longae, Radix Achyranthis Bidentatae, Herba Lycopodii Serrati

Rhizoma Curcumae Longae (RCL, *Curcuma longa* L. has antiplatelet and cholesterol-lowering as well as uterine stimulant effects [24]. Curcumin is a constituent of RCL frequently used as a food additive [24]. In rat retinal neurons, pretreatment with curcumin showed inhibitory effects against NMDA stimulation [25]. Both necrotic and apoptotic cell death induced by NMDA was decreased. The NMDA-elicited intracellular Ca^2+^ increase was also diminished. Thus, curcumin prevented the cascading events of Ca^2+^ overload from glutamate- (and NMDA)-associated excitotoxicity and subsequent neuronal death. Activation of NMDA-R in pathological conditions, *e.g*. ischaemia, is correlated with protein kinase activity [26]. While total levels of the NR1 subunit were unchanged, its phosphorylation was decreased in the presence of curcumin [25]. This latter finding suggests that in addition to its receptor blocking effects, curcumin also protects against abnormal NMDA-R activation in disease conditions. The evidence presented by Matteucci *et al*. supplemented earlier studies reporting the improvement of cognition and AD in animal models upon curcumin treatment [27,28].

Radix Achyranthis Bidentatae (RAB, *Achyranthes bidentata* Blume) has analgesic, anti-inflammatory and uterine stimulant effects [24]. A study has also reported that RAB promoted the regeneration of peripheral nerves [29]. Known constituents of RAB include aminobutyric acid and steroids such as ecdysterone and inoteosterone [24]. Aside from these constituents, a group has isolated what they called *Achyranthes bidentatae* polypeptides (ABPP) and reported neuroprotective effects [30,31]. Viability of rat hippocampal neurons challenged with NMDA was improved by ABPP pretreatment [30]. NMDA-induced intracellular Ca^2+^ increase was also diminished by ABPP but apparently with different selectivity toward NR2A and NR2B subunits [30]. NVP-AAM077, which has greater inhibitory effect on the NR2A than NR2B subunit, resulted in a diminished NMDA-induced Ca^2+^ response which was further suppressed upon addition of ABPP [30]. On the contrary, ABPP partially reversed the decrease in intracellular Ca^2+^ level resulted from prior addition of the selective NR2B subunit inhibitor Ro-256981 [30]. The authors interpreted these findings by suggesting that once the NR2A subunit was inhibited, the NR2B subunit would take over in facilitating the NMDA response, and as such, the observed ABPP effect was due to its selectivity toward the NR2A subunit [30]. Similarly, the NR2A subunit becomes predominant when the NR2B subunit was blocked, and thus the Ca^2+^-potentiating effect of ABPP reflected its NR2B selectivity [30]. However, the data may instead suggest ABPP having a synergistic or additive effect with NVP-AAM077 at the NR2A subunit as well as a competitively antagonistic effect with Ro-256981 at the NR2B subunit. This speculation, if proven true, would rule out the proposed NR2-subunit subtype selectivity of ABPP as Shen *et al*. has suggested [30]. With regards to the NMDA-induced Ca^2+^ responses as conducted in the same study [30], the selectivity of ABPP could be further elucidated if both the NR2A- and NR2B-subunit inhibitors are added followed by ABPP. In order to reveal the molecular interactions between the NR2 subunit subtypes and ABPP, structural biological techniques would be necessary. Intracellular Ca^2+^ increase precedes mitochondrial dysfunction and oxidative stress [32]. In another study by the same group, ABPP was found to prevent a drop in mitochondrial membrane potential, likely a result of blocked Ca^2+^ influx [31]. Besides, NMDA-induced production of reactive oxygen species (ROS) was also suppressed by ABPP [31]. Markers of apoptosis including Bax protein level and caspase-3 activity were lowered with prior addition of ABPP [31]. In rats having undergone ischaemia-reperfusion, brain infarct volume was smaller after ABPP treatment [31]. Although the exact identity of ABPP and its subunit selectivity is not known yet, the antioxidant and antiapoptotic potential of RAB in NMDA-associated neural diseases has been demonstrated.

Herba Lycopodii Serrati (HLS, *Lycopodium serratum* Thunb.) is commonly known as Chinese club moss [33] and possesses strong anti-acetylcholinesterase (AChE) activity [22]. Its AChE inhibitory effect makes HLS a suitable candidate for treatment in AD [23]. Isolation of active ingredients from HLS reveals the presence of huperzine A (HupA), which shows stronger anti-AChE effect than other conventional inhibitors such as rivastigmine and donepezil [34]. HupA is also found to have better bioavailability and permeability across the blood-brain barrier [22,23] and as such, was approved by the U.S. Food and Drug Administration to be marketed as a dietary supplement for use in AD [35]. Experimental studies have shown multiple neuroprotective effects of HupA, including decreased apoptotic gene expression, increased nerve growth factor secretion and reduced oxidative stress [36,37,38]. Particularly relevant to AD pathology is the inhibitory effect of HupA on cell death induced by amyloid-β peptides [39], the aggregation of which is integral to neurodegeneration. Existing data suggest that HupA exerts its effect not only by blocking AChE activity but also NMDA-R [33,40]. Zhang and Hu further showed that HupA blocked NMDA-induced current only whereas those induced by AMPA and kainite were unaffected in rat hippocampal neurons [41]. The concentration- current curve of NMDA with HupA showed a non-parallel shift, suggesting its uncompetitive antagonistic nature [41]. While the action of HupA on NMDA-R was not affected by simultaneous binding of the NTD (by Zn^2+^) and NR1 subunit (by glycine), addition of spermine (a positive modulator of NMDA-R at the NTD [2] increased the half-maximal inhibitory concentration of HupA [41]. It is possible that HupA competes with spermine at the same site as suggested by Zhang and Hu [41], or alternatively HupA binds to a different site than spermine but its presence renders the NMDA-R more activated and thus less subject to inhibition. Indeed, receptor binding assays in guinea pig cortical membranes showed that HupA did not alter NMDA binding to the ABD but that of MK-801 and phencyclidine was diminished [33], suggesting that HupA binds to the channel pore [33].

### 3.2. Radix Notoginseng

As stated earlier, the list of NMDA-R blockers include more than one herbal class according to the Chinese medical classification. In fact, herbs with apparently opposite therapeutic uses share inhibitory properties on the NMDA-R. As an example, Radix Notoginseng (RN, *Panax notoginseng* Burk.), known for its haemostatic effect, is a NMDA-R blocker much like herbs that promote circulation such as RCL. One of the RN constituents, notoginsenoside R1 (notoR1), has been shown to possess neuroprotective effects owing to its Ca^2+^-inhibitory, antioxidative and antiapoptotic actions [42]. In mouse cortical neurons, intracellular Ca^2+^ increase was diminished by notoR1, thereby reducing mitochondrial damage and subsequent events [42]. In the presence of notoR1, elevated level of ROS and the pro-apoptotic protein Bax was reversed, accompanied with increased amount of the antiapoptotic protein Bcl-2 back to the original level [42]. Unlike HupA (from HLS) which acts at the channel pore [33], notoR1 elicited its effects possibly at a site related to the NR2B subunit, as revealed by its selective expression on the cell [42]. Although its selectivity toward the NR2B over NR2A subunit has been shown, it remains unclear whether notoR1 interacts directly with the NR2B subunit. Further investigations of possible additive or antagonistic effects with other known NMDA-R modulators will be needed to determine the binding site of notoR1.

### 3.3. Radix Polygalae, Semen Zizyphi Spinosae, Radix et Rhizoma Valerianae

Radix Polygalae (RP, *Polygala tenuifolia* Willd.) has sedative effects and its inhibitory effect on AChE is shared also by HLS (discussed above) [22]. One of the active ingredients of RP is tenuifolin [43], which inhibits the secretion of amyloid-β peptides similar to HupA [44]. Another RP constituent, tenuigenin, has been shown to inhibit β-secretase activity, thereby reducing formation of amyloid-β peptides [44]. Using the crude extract of RP, Lee *et al*. showed that NMDA-induced death of rat cerebellar granular cells was inhibited [45]. As with most other herbs with NMDA-R blocking effects, RP resulted in diminished Ca^2+^ response and ROS production [45]. Glutamate- (and NMDA)-mediated excitotoxicity involves a positive-feedback loop that further enhances glutamate release in a retrograde signaling manner [1]. The finding that RP was able to suppress retrograde glutamate release adds to its protective effect against excitotoxicity [45]. Other RP constituents such as polygalasaponin that showed stimulatory effect on long-term potentiation and synaptic transmission provide additional evidence that RP may be an efficacious herb for use in AD and associated memory impairment [46].

Semen Zizyphi Spinosae (SZS, *Ziziphus jujube* Mill. var. *spinosa*), like RP, has sedative and also analgesic and antiseizure effects [24,47,48,49]. The CNS suppressant effect of SZS has been attributed to its constituents including spinosin and swertish [50]. The effect of methanol extract of SZS has been studied in detail in rat cerebellar granular cells [51]. NMDA-induced intracellular Ca^2+^ increase was almost completely abolished by SZS [51], a qualitatively stronger effect than other herbs that only partially diminished the Ca^2+^ response. The subsequent ROS production and cell death was also reduced by SZS. Similar to RP, SZS also suppresses glutamate release and may suggest additional protection for excitotoxicity [51].

Radix et Rhizoma Valerianae (RRV, *Valeriana officinalis*) possesses sedative, anti-inflammatory and anti-hyertensive properties [24,52,53]. In NMDA-stimulated mouse cortical neurons, cell death measured by lactate dehydrogenase (LDH)-associated membrane damage was abolished by an ethanol extract of RRV [52]. In the same study, kainate-induced cell death was marginally decreased only, suggesting the selective effect of RRV on NMDA-R over other glutamate receptor subtypes [52]. Receptor selectivity of RRV was further studied using its water extract and known constituents in rat cortical membranes [54]. Activity of RRV on glutamate receptor subtypes was complicated by dose-dependent variations. While RRV was inactive against the AMPA receptor, glutamate binding to NMDA-R was increased by a high concentration (10 mg/mL) of the RRV water extract [54]. Conversely, glutamate binding to kainate receptor was decreased by a low RRV concentration (0.05 mg/mL) [54]. The findings could be interpreted as a dual effect of RRV on different glutamate receptor subtypes—varied results on metabotropic glutamate receptors added to the complication [54]. When selected RRV constituents were examined, isoborneol but not valerenic acid demonstrated concentration-dependent effects on the glutamate receptors. At a low concentration (0.0008 mg/mL), isoborneol inhibited glutamate binding when AMPA and kainate receptors were activated [54]. An inhibitory effect on glutamate binding of NMDA-R was only observed when isoborneol was present at a high concentration (1 mg/mL) [54]. It is therefore likely that the NMDA-R-selective cellular effects reported by Jacobo-Herrera *et al*. were attributed to a high concentration of isoborneol or other RRV constituents yet to be identified in the ethanol extract [52]. Moreover, the use of whole RRV extract in targeting NMDA-R activity is cautioned due to the multi-faceted effect on all glutamate receptor subtypes, ionotropic and metabotropic. 

### 3.4. Ramulus Uncariae cum Uncis, Rhizoma Gastrodiae, Semen Cassiae, Rhizoma Acori

Ramulus Uncariae cum Uncis (RUU, *Uncaria rhynchophylla*) is used mainly to alleviate symptoms of tremor, dizziness and tinnitus [22,55]. In relation to AD pathology, amyloid-β formation was inhibited by RUU [56]. A methanol extract of RUU was found to reduce NMDA-induced rat hippocampal cell death [57], so as the water extract of RUU on mouse cortical neurons [58]. Diminished currents were observed under glutamate and NMDA stimulation, with a stronger RUU effect observed in the latter [57]. Interestingly, RUU concentration-dependently potentiated the AMPA-induced current [57]. Evidently, the whole RUU extract (this case in methanol) contains ingredients that could modulate glutamate receptors differently, a phenomenon also observed with RRV [54]. To further elucidate the NMDA-R effects of RUU, its chloroform-extracted alkaloid constituents (consisting of rhynchophylline, isorhynchophylline, *etc*.) were examined by Lee *et al*. [55]. Similar to the results of the RUU methanol extract, the alkaloid constituents inhibited NMDA-induced cell death [55]. Expression of the relevant genes was also suppressed in the presence of RUU alkaloids with the exception of the antiapoptotic Bcl-2 [55]. Although other modes of stimulation (*e.g*., by AMPA or kainate) were not investigated in the study, the results strongly suggest that the neuroprotective properties of RUU could be attributed to its inhibitory effect on NMDA-R. Moreover, the RUU alkaloids rhynchophylline and isorhynchophylline both inhibited NMDA-induced current [59]. The RUU-mediated enhanced AMPA-induced current may have possibly important functional implication, particularly since higher levels of the AMPA receptor than NMDA-R genes are expressed in the rat hippocampus [60].

Rhizoma Gastrodiae (RGas, *Gastrodia elata* Bl.) has sedative and antiseizure effects. One of its constituents, gastrodin, has been studied extensively for its CNS effects [61,62,63,64,65]. Gastrodin may also be indicated in Parkinson’s Disease as suggested by experimental evidence [66]. With regards to AD pathology, amyloid-β-induced rat hippocampal cell death was inhibited by RGas [67]. In rat cortical neurons, gastrodin inhibited cell death induced by both glutamate and NMDA [61]. Under hypoxic condition, the elevation of glutamate level was completely abolished by gastrodin, which also greatly improved post-ischaemic cell survival [61]. NMDA-induced glutamate release was also suppressed by gastrodin, suggesting its protective role in excitotoxicity similar to RP [45] and SZS [51]. Whether gastrodin (and RGas) also inhibits intracellular Ca^2+^ increase and ROS production like the other herbs (RP and SZS) remains to be investigated.

Semen Cassiae (SC, *Cassia obtusifolia* L.) is used for headache and dizziness in addition to its anti-hypertensive and antibiotic effects [24,68]. Evidence of memory improvement by SC has also been reported and attributed to its AChE inhibitory effect [69], thus suggesting its role in AD as well. Unlike other herbs (RCL, RAB, RN, RP, SZS) which directly diminished NMDA-induced Ca^2+^ responses, SC had no initial effect in mouse hippocampal cells [70]. However, the authors further analyzed the intracellular Ca^2+^ dynamics and discovered that SC elicited a complicated effect on the Ca^2+^ responses. Firstly, 1 µg/mL SC was shown to reduce the number of cells with elevated Ca^2+^ levels [70]. Two stages of Ca^2+^ responses were denoted, with both early-phase (within 10 min of NMDA addition) and late-phase (within 20–40 min of NMDA addition) intracellular Ca^2+^ increases inhibited by 1 µg/mL SC only [70]. The higher concentration of SC (10 µg/mL) was only effective at suppressing the late-phase NMDA-induced Ca^2+^ response [70]. Compared to the lower concentration, 10 µg/mL SC also appeared to be more harmful since Ca^2+^ removal was impaired in more cells [70]. In this experimental setting, 1 µg/mL SC demonstrated greater therapeutic potential than the higher concentration, and the ability to prevent late-phase Ca^2+^ overload is more relevant in apoptotic cell death in AD [71]. Indeed, mitochondrial dysfunction and cell death were both suppressed by SC (1 µg/mL). The current evidence supports the neuroprotective action of SC, but its inability to readily inhibit Ca^2+^ increase and the inverse concentration-dependent effect raise questions as to whether interaction with the NMDA-R takes place. The possibility that SC affects NMDA-mediated signaling remains.

Rhizoma Acori (RA, *Acorus tatarinowii* Schott) belongs to a different Chinese medical category as other sedative herbs probably due to its anecdotal effect to restore consciousness from coma [24]. Different effects of RA extracts have been reported depending on the extraction method used [24]. For example, the antiseizure effects of RA were found in its water extract only, whereas the sedative effect was demonstrated in both water and methanol extracts [24]. On the other hand, the neuroprotective effect of RA has been attributed to its methanol extract and essential oil [72,73]. Studies have that asarone, an essential oil constituent of RA, possess antioxidant, neuroprotective and memory-improving effects [74,75]. Asarone extracted directly from RA have been shown to inhibit NMDA-induced death of rat cortical neurons [76]. On the contrary, cell death induced by AMPA or kainate was not affected by asarone, suggesting its selective NMDA-R action [76]. Receptor binding assays further revealed that asarone interacted with the channel pore but not the glycine-binding site on the NR1 subunit [76]. Another study by the same group had earlier reported that the RA methanol extract interacted with both channel pores and NR1 subunit [72]. These findings indicate the overall neuroprotective effect of RA may be attributed to activity at different binding sites on the NMDA-R.

### 3.5. Radix Glycyrrhizae, Radix Ginseng

Radix Glycyrrhizae (RGly, *Glycyrrhiza uralensis* Fisch.) possesses a multitude of effects including antidiuretic, anti-inflammatory and antispasmodic [24,77]. An active ingredient of RGly, glycyrrhizic acid (GlyA), has also been used in hepatitis treatment [78]. In rat cerebellar granular neurons, GlyA suppressed glutamate-induced cell death [77] by inhibiting the activity of the apoptotic enzyme caspase-3 [77]. Upstream of apoptosis, glutamate-induced Ca^2+^ increase was also diminished in the presence of GlyA [77]. More pronounced inhibition of the Ca^2+^ response was observed when GlyA and APV were added together [77]. Since other NMDA-R antagonists were not examined, further studies of molecular interactions will be necessary to ascertain the active site of GlyA. Nevertheless, this latter finding may suggest that GlyA exerts its action at a site away from the glutamate-/NMDA-binding site, which is bound by APV.

Radix Ginseng (RGin, *Panax ginseng*) has immunostimulant, ionotropic, neurotropic, neuroprotective, cognitive-improving and memory-enhancing effects, and is probably one of the most studied Chinese herbs [24,79]. Only the NMDA-R-associated effects of RGin will be considered here. For a more extensive overview of RGin and its CNS effects, readers are referred to Jesky and Hailong [23]. The neural effects of RGin have been observed using ginseng total saponins (GinTS) and their constituent ginsenosides [23]. In rat hippocampal cells, GinTS diminished primarily NMDA-induced current and Ca^2+^ response [80], but also those induced by AMPA and kainate [80]. Several constituents of GinTS, including Rb1, Rh2 and Rg3, were found to elicit Ca^2+^-inhibitory effect against NMDA stimulation [80]. Both Rb1 and Rh2 exerted a smaller inhibitory effect on Ca^2+^ increase than Rg3. In another study using mouse mesencephalic dopaminergic neurons, Rb1 was reported to improve cell survival after glutamate-induced neurotoxicity [81]. Whether Rb1 treatment also diminished NMDA-, AMPA- or kainate-induced neurotoxicity was not investigated, thus raising the possibility that Rb1 may have a better selectivity against other glutamate receptor subtypes. It is also possible that responses of different types of neurons (*i.e.*, dopaminergic vs. glutaminergic) may depend on the individual ginsenosides. According to Kim *et al*., Rg3 displays the strongest Ca^2+^-inhibitory effect among all ginsenosides examined [80]. The receptor-binding characteristics of Rg3 was further elucidated. Rg3 caused a nonparallel shift of the NMDA concentration-response curve [79], suggesting the ginsenoside acts uncompetitively at the NMDA-R [79]. Pretreatment of Rg3 largely diminished the NMDA-induced Ca^2+^ response, compared to the relatively smaller Ca^2+^ inhibitory effect of MK-801 pretreatment [79]. The authors thus concluded that Rg3 and MK-801 bind to different sites on the NMDA-R, since the latter drug requires prior channel-opening for its action. It was then revealed, from parallel shifts of concentration-response curves, that Rg3 competes with glycine for its binding site [79]. It was mentioned earlier that Rh2 elicits a weaker Ca^2+^-inhibitory response than Rg3 [80]. This may be attributed to the racemic mixture of Rh2 which attenuated its action, as implied by a study using the active *S*-isomer of Rh2 (SRh2) [82]. Similar to Rg3, SRh2 also acted as an uncompetitive NMDA-R antagonist but at different sites, as suggested by the additive effect in the presence of both [82]. Unlike Rg3 which acts at the glycine-binding site, SRh2 is antagonistic at the polyamine- (or spermine-) binding site [82]. The inhibitory effect of NMDA-induced Ca^2+^ response and current was diminished in the presence of spermine [82], which is a positive modulator of NMDA-R, a result similar to that observed with HupA [41]. Despite functional data suggesting the sites of action of Rg3 (at the glycine-binding site) and of SRh2 (at the polyamine-binding site), stronger evidence may be provided when receptor-binding assays are conducted, as in the case with HupA.

### 3.6. Folium Gingko

Besides its use as an expectorant in Chinese medical practice, Folium Gingko (FG, *Ginkgo biloba* L.) has demonstrated effects in cognitive-improvement of AD patients [83,84] and in vascular diseases [85]. The underlying effect is likely owing to its inhibition on amyloid-β formation. Ginkgolide B and bilobalide are two FG constituents showing neuroprotective effect after glutamate stimulation [86]. Bilobalide has also been shown to inhibit downstream signaling of NMDA in rat hippocampal cells [87]. EGb761, a FG extract containing flavonoids and terpenes, have displayed antioxidant and antiapoptotic effects [88]. Specific NMDA-R binding site(s) of FG have not been located, but a group has found that FG extract particles of smaller than 100 nm (as compared to particles in the micrometer range) may show improved water solubility, delivery across the blood-brain barrier, and efficacy in inhibiting the NMDA-induced current [85].

### 3.7. Radix Scrophulariae

Radix Scrophulariae (RS, *Scrophularia ningpoensis* Hemsl.) has shown antibacterial, antifungal as well as sedative effects [24]. Experimental findings have revealed the neuroprotective effect of RS in glutamate-stimulated rat cortical neurons [89]. In a methanol extract of RS, iridoids have been identified as the active class of compounds with neuroprotective effect [90]. More detailed examination was performed on two of the iridoids, 8-*O*-E-p-methoxycinnamoylharpagide (MHarp) and harpagide (Harp) [91]. While Harp suppressed both NMDA- and kainate-induced rat cortical cell death, MHarp did so more selectively against NMDA stimulation [91]. Intracellular Ca^2+^ increase and ROS production, two consequences of NMDA-R-mediated Ca^2+^ signaling, were also prevented by MHarp but not Harp in the presence of glutamate [91]. The present evidence suggests that MHarp may exert its action via NMDA-receptor blockade, but the effect may also arise from AMPA-receptor antagonism which the said study did not examine [91]. Moreover, while the neuroprotective effect of MK-801 was shown along with that of MHarp, the two were not applied together to investigate possible additive or antagonistic effect. The use of known NMDA-R antagonists simultaneously with MHarp and other RS iridoids will reveal more information on their receptor-binding characteristics.

## 4. Conclusions

The research on herbs with NMDA-R effects has provided valuable information on cellular function and intracellular signaling mechanisms. One could extrapolate from these findings and proposes possible clinical applications of the individual herbs. However, in order to better supplement conventional western medicines in targeting the receptor entity, namely the activity of NMDA-R, it is necessary to have greater understanding of molecular interactions between the herbs and the various binding sites on the receptor subunits. Except for a small number of herbs and their constituents, e.g. HupA, GlyA, RRV, much about receptor-binding and -selectivity is yet to be determined. On another note, a majority of the studies reporting the herbal effect on NMDA-R activity used cells related to memory (*i.e*., from hippocampus) and cognition (*i.e*., from cortex). The choice of cell types is rational since NMDA-R modulation is strongly correlated with the treatment of AD, but future investigations may also consider examining cells from other brain areas so that potential adverse effects of the target herbs and herbal constituents will not go unnoticed. Lastly, evidence of neuroprotection observed in several herbs may suggest their action on the NMDA-R as well. It will be of interest to study the receptor-binding characteristics of these herbs and their constituents.
